# Wide-Ranging Analysis of MicroRNA Profiles in Sporadic Amyotrophic Lateral Sclerosis Using Next-Generation Sequencing

**DOI:** 10.3389/fgene.2018.00310

**Published:** 2018-08-14

**Authors:** Bruna De Felice, Francesco Manfellotto, Giuseppe Fiorentino, Anna Annunziata, Elio Biffali, Raimondo Pannone, Antonio Federico

**Affiliations:** ^1^Department of Environmental, Biological and Pharmaceutical Sciences and Technologies, University of Campania “Luigi Vanvitelli”, Caserta, Italy; ^2^Division of Physiopathology, Monaldi Hospital, Naples, Italy; ^3^Zoological Station Anton Dohrn, Naples, Italy; ^4^Institute of Genetics and Biophysics “Adriano Buzzati Traverso”, National Research Council, Naples, Italy

**Keywords:** amyotrophic lateral sclerosis, microRNAs, neurodegenerative diseases, NGS data analysis, sporadic ALS

## Abstract

MicroRNA (miRNA) has emerged as an important regulator of gene expression in neurodegenerative disease as amyotrophic lateral sclerosis (ALS). In the nervous system, dysregulation in miRNA-related pathways is subordinated to neuronal damage and cell death, which contributes to the expansion of neurodegenerative disorders, such as ALS. In the present research, we aimed to profile dysregulation of miRNAs in ALS blood and neuromuscular junction as well as healthy blood control by next-generation sequencing (NGS). The expression of three upregulated miRNAs, as miR-338-3p, miR-223-3p, and miR-326, in the ALS samples compared to healthy controls, has been validated by qRT-PCR in a cohort of 45 samples collected previously. Bioinformatics tools were used to perform ALS miRNAs target analysis and to predict novel miRNAs secondary structure. The analysis of the NGS data identified 696 and 49 novel miRNAs which were differentially expressed in ALS tissues. In particular, in neuromuscular junction the differential expression of miR-338-3p, which we previously found upregulated in different types of ASL tissues, miR-223-3p, and miR-326 was elevated compared to normal control. ALS miRNAs gene target were significantly involved in neuronal related pathway as BDFN1 and HIF-1genes. This study presents the direct experimental evidence that, overall, miR-338-3p is highly expressed in ALS tissues including neuromuscular junction characterizing ALS from normal tissues. Beside, our analysis identified, for the first time, novel miRNAs highly expressed in ALS tissues. In conclusion, the results indicate that miRNAs has an important role in the diagnosis and treatment of ALS.

## Introduction

Amyotrophic lateral sclerosis (ALS) is an escalating neurological disease that mainly involves the degeneration of cortical and spinal motor neurons, leading to paralysis and death within 3–5 years from when the first symptoms appear. More than 90% of cases are evaluated sporadic, since they occur randomly, without a clear family linkage, whereas the remains are inherited, usually as an autosomal dominant trait but they are also inherited as an autosomal recessive or X-linked trait (Turner et al., 2013). To define ALS cases as familial or sporadic resting on family history should not be considered in total terms. All cases of ALS, including sporadic ALS, underlie a combination of genetic and environmental factors, which are not directly causative; however they act as trigger factors in genetically predisposed individuals. Mutations in several genes account for familial ALS and contribute to the development of a small percent (11%) of sporadic ALS. Genes mutations associated with ALS pathology involve superoxide dismutase 1 (SOD1), fused in sarcoma (FUS), TAR DNA-binding protein (TARDBP) and a hexanucleotide repeat expansion on chromosome 9 in open reading frame 72 (C9ORF72). Such gene mutations account for only 11% of sporadic ALS cases and 70% of familial ALS (Renton et al., 2014). The pathogenesis is still unclear but it is believed that multiple mechanisms are involved, including glutamate-mediated excitotoxicity, axonal transport deficits, oxidative stress, protein misfolding and mitochondrial dysfunction (Wijesekera and Leigh, 2009). Even aberrant RNA processing plays a role in motor neurons degeneration (Droppelmann et al., 2014); indeed, several genes associated with ALS are related to RNA metabolism, such as FUS (Kwiatkowski et al., 2009), TARDBP gene (Strong et al., 2007).

Only in the last decade were miRNAs, small non-coding RNA molecules of 21–22 nucleotides, discovered. miRNAs inhibit mRNA translation of target genes or induce its degradation through imperfect complementarity with its target site, providing a new and fine post-transcriptional gene expression regulation mechanism, which is central to a wide domain of cellular processes, such as proliferation, apoptosis and physiological processes, including development and function of central nervous system (Kloosterman and Plasterk, 2006).

miRNA genes are scattered throughout all genome or organized in gene clusters. Biogenesis of miRNAs is a multistep process which starts in the nucleus and ends in the cytoplasm with subsequently many post-transcriptional modifications. miRNAs have been discovered in plants, animal and viruses, and are involved in all cellular processes (Ardekani and Naeini, 2010). In recent years, scientific community has clarified in part the role of miRNAs in cellular function. They are estimated to target and regulate the expression of more than 30% of mammalian genome (Lewis et al., 2005). In humans, more than 2000 *miR* genes have been identified by experimental proof, even if the total number is not defined. miRNA deregulation is involved in different human diseases including cancer, autoimmune disorders, and pollution (Erson and Petty, 2008). In several neurodegenerative diseases, such as Parkinson’s disease (Minones-Moyano et al., 2011), Huntington’s and Alzheimer’s disease (Borovecki et al., 2005), dysregulation of miRNA expression levels have been identified. Several studies report aberrant miRNAs expression in ALS patients too and suggest that miRNAs play a role in the development and progression of disease by regulation of inflammation (Butovsky et al., 2012), neurofilament (Campos-Melo et al., 2013), mitochondrial function (Russell et al., 2012), endoplasmic reticulum stress, and glutamate transport (De Felice et al., 2012). The identification of deregulated miRNAs in ALS patients might help to elucidate pathogenic mechanisms of sALS, allow earlier diagnosis and provide targets for innovative therapies. Although research on miRNAs is only at the beginning, it is already clear that they play an important role in a broad-spectrum of diseases, including ALS.

In the present research we aimed to characterize dysregulation of miRNAs in ALS blood and neuromuscular junction as well as healthy blood control, and the miRNAs were profiled by NGS. The analysis of the NGS data identified 696 and 44 novel miRNAs, which were expressed in ALS tissues. Three upregulated and two upregulated miRNAs were selected for validation by qRT-PCR. Here, in neuromuscular junction the differential expression of miR-223-3p, miR-338-3p, and miR-326 was significantly elevated compared to normal control. Moreover, novel miRNAs were identified here in all ALS samples. In particular, in ALS neuromuscular junction a novel miRNA, which has coordinates chr5:93905132-93905758, was highly expressed compared to control samples. Significantly, NGS analysis has identified novel miRNA signature and strictly confirmed miR-338-3p as miRNA expressed in all ALS samples, distinguishing normal from neurodegenerative samples. This study provides a starting point for future studies to the comprehension of the roles of novel miRNAs in ALS pathological process.

## Materials and Methods

### Patient Details

Peripheral blood and neuromuscular junction from a total of 70 patients with sporadic ALS (*n* = 45), and healthy controls (*n* = 25) was obtained (see **Supplementary Table S1**). Blood samples from healthy controls and from patients with ALS were obtained from the Monaldi Hospital, Naples, Italy and neuromuscular junction from ALS patients were purchased from Edinburgh Tissue Bio Bank. Detailed patient characteristics are listed in **Supplementary Table S1**. ALS patients have been diagnosed as clinically definite sALS with the support of electroneuromyography (ENMG). The onset of the disease was 1–2 years before diagnosis. The mean age of patients and controls was 62 ± 10 years. This study was approved by the institutional review boards of University of Naples II, Italy and the study was performed in accordance with the Declaration of Helsinki. Written informed consent was obtained from all patients participating in the study.

We analyzed the expression of miRNAs in all samples from patients with sporadic ALS and healthy controls either by NGS or by RT-qPCR or by both methods.

### RNA Extraction From Blood and Neuromuscular Junction

RNA extraction was performed as in De Felice et al. (2014). Briefly, we obtained 10 μg of total RNA from peripheral blood samples drawn from patients and controls in the morning. Trizol (Invitrogen, no. 15596-026) method has been used for isolation and purification of RNA from whole blood and from neuromuscular junction samples. RNA was isolated including a DNase digestion step.

These standardized RNA isolation procedures guarantee high-quality RNA. Using the Agilent 2100 Bioanalyzer platform (Agilent Technologies) RNA samples were quality-checked documenting the identification of 18-S and 28-S ribosomal RNA (rRNA) peaks. The yields were 8–15 μg, and the RNA Integrity Number (RIN) was between 7.2 and 10. Such results fulfilled published data, reporting that RNA with a RIN > 6 is of sufficient quality for miRNA microarray experiments as well as gene and miRNA expression-profiling experiments.

### Small RNA Sequencing Libraries Generation

Small RNA libraries were generated using RNA extracted from three cohorts of patients across three different conditions. Specifically, we sequenced the small RNAs from neuromuscular junctions of three patients affected by SLA, blood samples from three SLA affected patients and blood samples from two healthy patients used as controls.

We performed the enrichment of small RNA fraction from total RNA utilizing the Magnetic Bead Cleanup Module (Life Technologies, Carlsbad, CA, United States). Using Ion Total RNA-Seq kit v2 Life Technologies, Carlsbad, CA, United States, 50 ng of enriched small RNA sample was ligated to proper adapters and reverse transcribed to cDNA.

cDNA samples were size-selected from 50 to 300 nt (Magnetic Bead Cleanup Module, Life Technologies, Carlsbad, CA, United States), and PCR amplified using 5′ primers containing a unique index barcode (Ion XpressTM RNA-Seq Barcode 1–16 Kit, Life Technologies, Carlsbad, CA, United States). We assessed the yield and size distribution of the small RNA libraries using the Agilent 2100 Bioanalyzer^TM^ instrument and the Agilent HS DNA kit.

We amplified similarly pooled libraries on Ion Sphere^TM^ Particles (ISPs) provided by the Life Technologies Ion PI Hi-Q Chef kit. All the steps to perform the emulsion PCR and the loading of the pooled libraries in the Life Technologies ION PI Chip v3 were performed automatically on Life Technologies ION Chef Instrument. Eight libraries were pooled in one chip. Sequencing was performed with Life Technologies ION TOTAL RNA-SEQ KIT V2 on Life Technologies ION Proton Sequencer. All data has been assigned ArrayExpress accession E-MTAB-7073^1^.

### Data Analysis

Reads underwent to quality control and were and removal of 3′ adapters using Cutadapt tool (Martin, 2011) and FasQC (Andrews, 2010). Detection of both known miRNAs and prediction of novel ones, was performed using miRanalyzer version 0.3 (Hackenberg et al., 2011), which is based on well known ultrafast short-read aligner Bowtie (Langmead, 2010). Specifically, reads were aligned toward miRBase v.20. In order to identify several types of small non-coding RNAs. After that, remaining reads were aligned toward other sncRNAs libraries such as piRNABank, RFAM and tRNAs downloaded from UCSC Genome Browser. For each genomic entry we evaluate the expression as the number of mapped reads in each sample. Zero-counts and low counts reads were filtered out setting at least five reads as minimum cut-off in all conditions. All the above-mentioned steps were performed using iMir (Giurato et al., 2013). iMir integrates several R-packages in a useful GUI (Graphical User Interface). Upper quartile was used to normalize counts across different samples. Using the Bioconductor package NOISeq (Tarazona et al., 2011) differential expression analysis between conditions has been performed. The selection of differentially expressed miRNAs was done on the basis of posterior probability, PP, with pp ≥ 0.8. To identify the protein-coding transcript potentially regulated by differentially expressed miRNAs, we carried out a Target prediction using TargetScan and Pictar tools (Krek et al., 2005; Lewis et al., 2005; Grimson et al., 2007). We considered significant target as previously reported (Jansen et al., 2011). To build the protein–protein interaction (PPI) network and the functional annotation String database was used and to perform Gene ontology and the functional annotation (Szklarczyk et al., 2017). All the exploratory data analyses as well as graphs were complemented through the building of customized R scripts.

For each novel miRNAs the secondary structure was predicted using RNAstructure (Reuter and Mathews, 2010) with default parameters.

### Validation of miRNAs by qRT-PCR

Validation of miRNAs expression was performed according to De Felice et al. (2012). qRT-PCR was performed using a TaqMan miRNA assay kit (Applied Biosystems, Foster City, CA, United States) to quantify miRNA expression levels. miRNAs were reverse transcribed using TaqMan MicroRNA RT kit (Applied Biosystems) and real-time PCR was performed using an 7500 Fast Real-Time PCR system (Applied Biosystems, Foster City, CA, United States), following the D-D-Ct method. RNU6B was utilized for an endogenous reference. All the data were calibrated by the universal reference data. The results are mean ± SD of at least three separate experiments, measuring each parameter by triplicate (*n* = 3). We used one way analysis of variance (ANOVA) to test statistical significant differences, and Student–Newman–Keuls test when the *F*-value was significant. *p*-Value less than 0.05 (^∗^) was considered statistically significant.

## Results

### High-Throughput Sequencing of the Small RNA Fraction

The sequencing of the small RNA fraction was performed on three cohorts of patients across three different conditions. Specifically, we sequenced the small RNAs from neuromuscular junctions of three patients affected by ALS, blood samples from three ALS affected patients and blood samples from two healthy patients used as controls.

**FIGURE 1:**
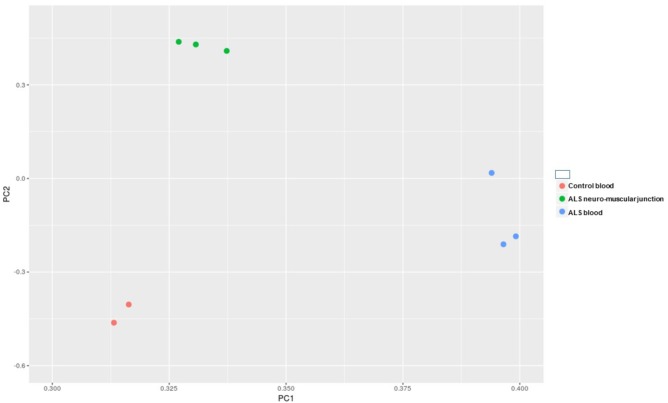
Principal component analysis (PCA) of all of the samples underwent to small RNA-Seq analysis.

After the removal of adapters and the quality check, we obtained in average 3.88 million reads per sample, ranging from 2.46 to 5.92. A length distribution analysis was performed on each sample (**Supplementary File S1**). As shown in **Supplementary File S1**, in the neuromuscular junction samples we obtained a predominant peak around 22 bp (belonging to the miRNA fraction, which we are interested in), and another peak around 32 bp (belonging to other species of small RNAs). In all the remaining samples we observed the miRNAs peak considerably higher in respect of the other peak. After the alignment and the counting of the cleaned reads, we performed an exploratory data analysis. The principal component analysis (PCA) has shown (**Figure 1**) that the replicates within the same condition are well grouped and separated by the other samples.

### Identification of Dysregulated miRNAs Across the Conditions

**FIGURE 2:**
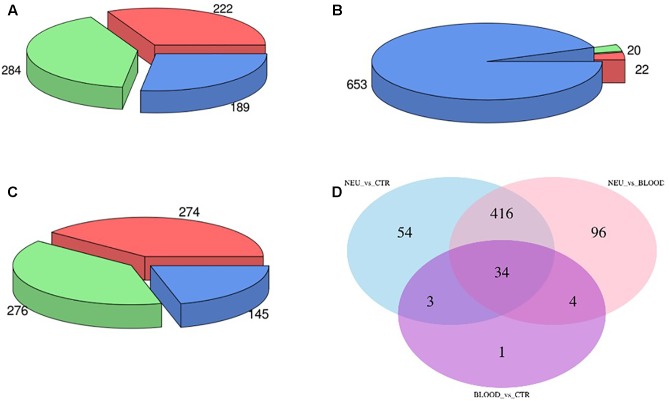
Panel **(A)** shows the amount of dysregulated miRNAs in the comparison between the neuromuscular junctions and the control blood samples. Panel **(B)** shows the amount of dysregulated miRNAs in the comparison between the blood samples from ALS affected patients and the neuromuscular junctions. Panel **(C)** shows the amount of dysregulated miRNAs in the comparison between the blood samples from ALS affected patients and the control. Panel **(D)** shows the Venn diagram of dysregulated miRNAs in all the three comparisons.

The computational analysis of our datasets allowed us to obtain an accurate expression profiling of the expressed miRNAs. Overall, we found that 696 miRNA were expressed across all analyzed samples. In order to assess the fraction of miRNAs or miRNA families significantly dysregulated in both neuromuscular and blood samples from ALS affected patients compared to the controls we carried out a differential expression analysis. In particular, in the comparison between the neuromuscular junctions and the control blood we obtained that 284 miRNAs were upregulated and 222 were downregulated (**Figure 2A**). Only 20 miRNAs were upregulated and 22 were downregulated comparing between ALS blood samples and the control blood (**Figure 2B**). To increase the validity of our analysis and exclude dysregulated miRNAs only based on the different tissues instead of the pathological condition, we performed a differential expression analysis between the neuromuscular junctions and blood by ALS affected patients. Such analysis demonstrated that 276 miRNAs were upregulated and 274 were downregulated in the neuromuscular junctions compared to ALS blood (**Figure 2C**). In order to identify tissue-related differentially expressed miRNAs, we intersected dysregulated miRNAs from each of the three evaluations (**Figure 2D**).

**FIGURE 3:**
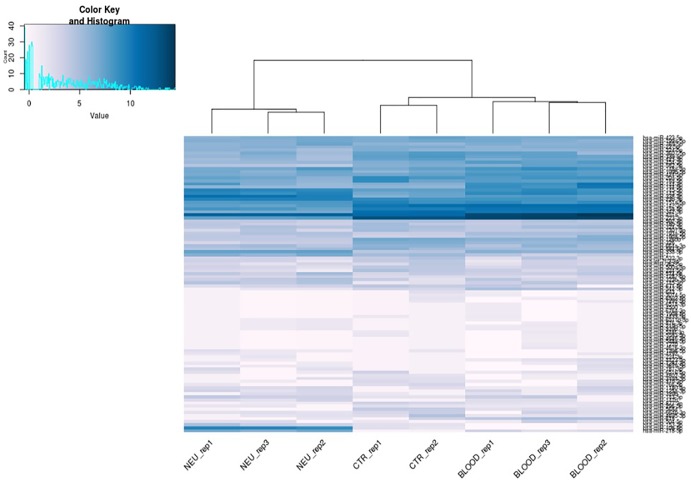
Heat map of differentially expressed microRNAs. *Blue* represents upregulation and *white* represents downregulation.

Specifically, we found that the dysregulation of 416 miRNAs could be tissue specific rather than pathological condition, and not significant. Such miRNAs have been excluded from the subsequent analysis. Indeed, 91 differential expressed miRNAs have been considered suitable for the analysis from the comparison between the neuromuscular junction and control blood. Expression profiles of differential expressed miRNAs are shown in the heatmap in **Figure 3**. As shown in the heatmap, all the expressed miRNAs displayed significantly different expression profiles among different tissues. In fact, the miRNAs expressed in the neuromuscular junctions are clearly clustered regarding all the other samples.

**Supplementary File S2** shows the significant differential miRNAs expression (log2 Fold-Change and posterior probability) in three group comparisons (ALS blood samples group vs. blood control group, ALS neuromuscular junction group vs. ALS blood samples group or blood control group).

**FIGURE 4:**
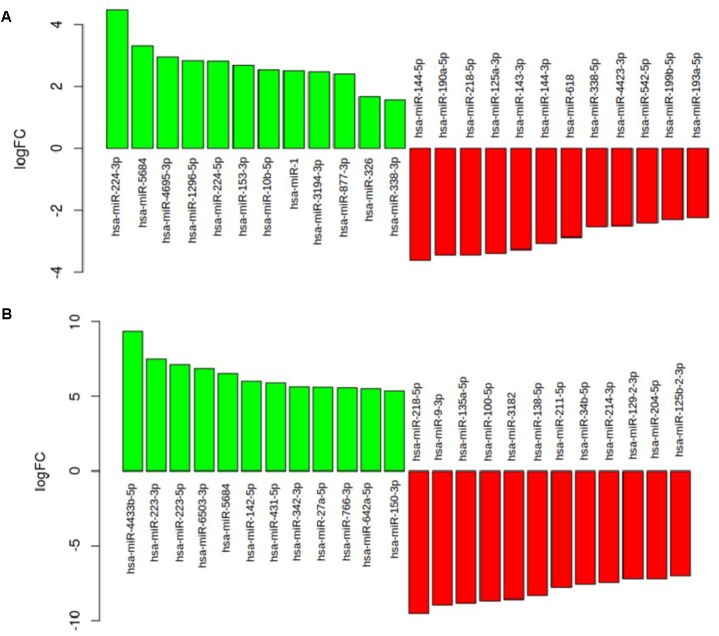
**(A)** The barplot show the most deregulated miRNAs in the comparison between the ALS blood and the control blood. **(B)** The barplot show the most deregulated miRNAs in the comparison between ALS neuromuscular junction and the control blood.

As shown in **Figure 4B**, in the comparison between the neuromuscular junction and the control blood the most upregulated miRNAs were hsa-miR-223-3p (logFC = 7.48; *p* = 0.99), hsa-miR-5684 (logFC = 6.52; *p* = 0.99), hsa-miR-338-3p (logFC = 5.21; *p* = 0.99), hsa-miR-342-3p (logFC = 5.65; *p* = 0.99), and hsa-326-miR-326 (logFC = 4.8; *p* = 0.99). The most downregulated miRNAs were hsa-miR-218-5p (logFC = -9.56; *p* = 1), hsa-miR-10b-5p (logFC = -11.08; *p* = 1), and hsa-miR-338-5p (logFC = -4.46; *p* = 1). As shown in **Figure 4A**, in the comparison between the SLA blood and control blood the most upregulated miRNAs were hsa-miR-224-3p (logFC = 4.48; *p* = 0.82), hsa-miR-5684 (logFC = 3.32; *p* = 0.81), hsa-miR-4695-3p (logFC = 2.96; *p* = 0.80), and hsa-miR-1296-5p (logFC = 2.83; *p* = 0.81). The most downregulated were hsa-miR-144-5p (logFC = -3.61; *p* = 0.9), hsa-miR-190a-5p (logFC = -3.43; *p* = 0.8), hsa-miR-218-5p (logFC = -3.43; *p* = 0.92), and hsa-miR-125a-3p (logFC = -3.38; *p* = 0.86).

### Validation of Dysregulated miRNA Candidates in an Independent Cohort of ALS Blood and Neuromuscular Junction Samples

**FIGURE 5:**
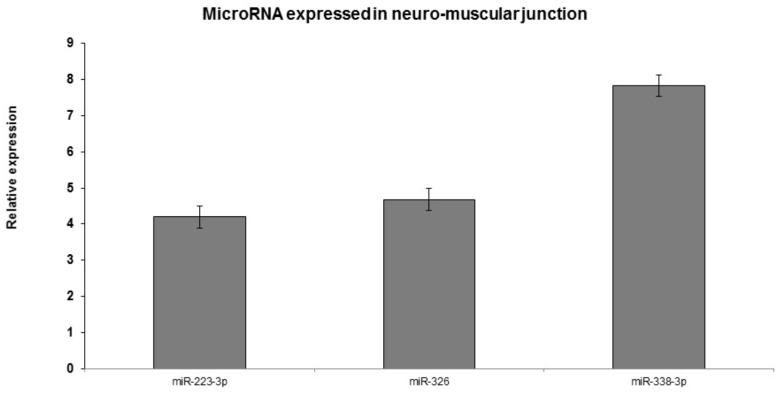
Real-time PCR analyses of significantly dysregulated miRNAs in ALS blood and neuromuscular junction compared to the control blood. *p* < 0.005. qRT-PCR validation of miRs: representative miRNAs were validated using qRT-PCR. Similar direction of effect was observed as seen in the next-generation sequencing with statistical significance of *p* < 0.05.

The expression level of the 10 selected miRNAs were additionally confirmed in an independent and novel cohort of 45 ALS samples and 25 healthy controls by qRT-PCR. In particular, in neuromuscular junction the expression of miR-223-3p, miR-338-3p, and miR-326 was elevated in 97% patients compared to blood samples with statistical significance (*p*-value <0.01) (**Figure 5**).

### Functional Annotation of Target mRNAs

To assess the mRNA whose expression is controlled by miRNAs found dysregulated in our analysis, we performed a target prediction analysis intersecting the results from both TargetScan and Pictar. According to this approach, we obtained a list of 601 protein encoding genes, which, with high confidence, are target by at least one of deregulated miRNAs identified in the previous analysis. In order to strengthen our findings and select a subgroup of target genes strictly related to ALS, we addressed which of these protein coding genes were directly associated with ALS by previous genome-scale experiments and whether such genes could be selected as potential target. Specifically, inspecting our cohort of genes with OpenTarget database, we assessed that 94 out of 601 were annotated as potentially associated to the disease and estimated as pharmacologic targets. Since these genes are particularly interesting for their association to ALS we performed a functional annotation procedure.

In order to clarify the impact of such genes on a molecular and cellular level and to better understand which biological function is mainly affected by their dysregulation caused by perturbed miRNAs, we constructed a PPI network.

**FIGURE 6:**
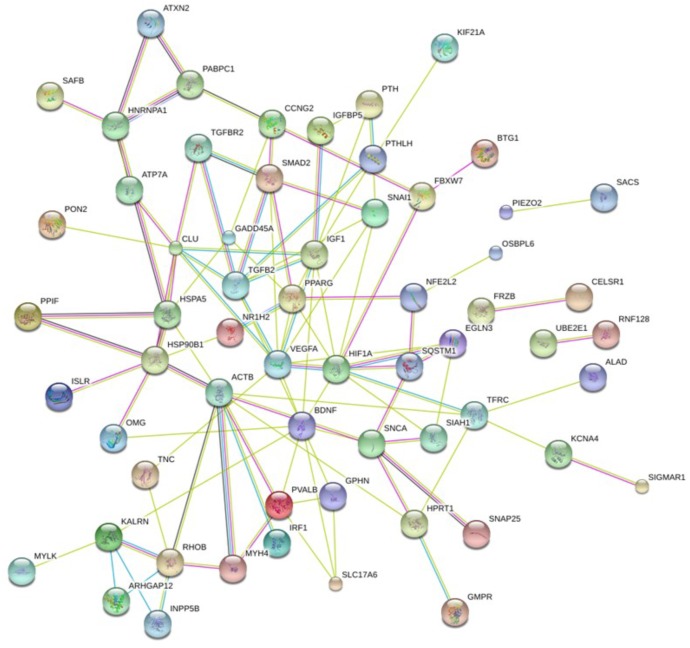
Network showing protein–protein interaction (PPI) among identified target genes.

**FIGURE 7:**
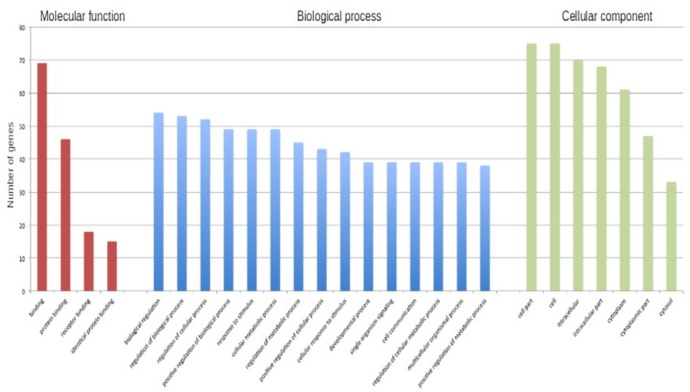
The barplot shows the number of ALS-associated genes enriched in each of reported GO term.

In particular, we submitted the target genes to the KEGG pathway and Gene Ontology tools both implemented in String database. As shown in **Figure 6**, one of the central node is BDFN1, brain-derived neurotrophic factor, which is implicated in metabolic syndrome and neurodegenerative diseases like Huntington’s, Alzheimer’s, and Parkinson’s disease and depression (Motamedi et al., 2017). BDFN1, VEGFA, ACTB, and KARLN are involved in such pathway. Moreover, HIF1 pathway has been found significantly perturbed (FDR = 0.0081). HIF1A, VEGFA, EGLN3, TFRC, and IGF1 essentially contributed to the deregulation of such pathway. Regarding the Gene Ontology analysis, as shown in **Figure 7**, we obtained very general terms enriched for the molecular function and biological process categories. In fact, the molecular function category indicates mainly a binding function for genes of interest while for the biological process category we observed that genes are involved in regulation of generic biological, cellular, and metabolic processes. The Cellular component category indicates that target genes associated to ALS are functionally active mainly in the cytoplasm and in the perinuclear region of the cell and in the axonal region of the neuronal cells.

Even though this subset of genes showed a significant enrichment for GO terms describing general molecular and biological features, as reported in **Figure 7**, we observed a statistically significant enrichment of a subgroup of genes involved in several processes of the neuronal compartment as well as neuron differentiation, morphogenesis and development. All the biological processes related to the neural compartment in which this set of genes is involved are listed in **Table 1**.

**Table 1:** Functional and pathway analyses of the genes in the network.

Term	Pathway	Genes	FDR
GO.1901214	Regulation of neuron death	ATP7A, BDNF, BTBD10, CLU, EGLN3, FBXW7, HIF1A, SIGMAR1, SNCA, TGFB2	6.41e-05
GO.0022008	Neurogenesis	ACTB, ATXN2, CELSR1, CLU, HIF1A, HPRT1, IGF1, KALRN, MATN2, NAP1L1, PPARG, RHOB, SIAH1, TGFB2, TNC, VEGFA	0.0132
GO.0043523	Regulation of neuron apoptotic process	ATP7A, BDNF, EGLN3, FBXW7, HIF1A, SIGMAR1, SNCA, TGFB2	0.000471
GO.0031175	Neuron projection development	ACTB, ATXN2, HPRT1, KALRN, MATN2, OMG, RHOB, SIAH1, SNAP25, TGFB2, TNC, VEGFA	0.00264
GO.0048667	Cell morphogenesis involved in neuron differentiation	ACTB, ATP7A, BDNF, HPRT1, KALRN, MATN2, RHOB, SIAH1, SNAP25, TGFB2, VEGFA	0.00204
GO.0048666	Neuron development	ACTB, ATXN2, HPRT1, KALRN, MATN2, OMG, RHOB, SIAH1, SNAP25, TNC, VEGFA	0.0259
GO.0061564	Axon development	ACTB, BDNF, KALRN, MATN2, RHOB, SIAH1, SNAP25, TGFB2, TNC, VEGFA	0.00558
GO.0007409	Axonogenesis	ACTB, BDNF, KALRN, MATN2, RHOB, SIAH1, SNAP25, TGFB2, VEGFA	0.0141
GO.0048812	Neuron projection morphogenesis	ACTB, ATXN2, HPRT1, KALRN, MATN2, RHOB, SIAH1, SNAP25, TGFB2, VEGFA	0.00834
GO.0007411	Axon guidance	ACTB, BDNF, KALRN, MATN2, RHOB, SIAH1, TGFB2, VEGFA	0.0163
GO.1902692	Regulation of neuroblast proliferation	BDNF, HIF1A, VEGFA	0.0174
GO.1901215	Negative regulation of neuron death	ATP7A, BDNF, BTBD10, HIF1A, SNCA	0.0297
GO.2000177	Regulation of neural precursor cell proliferation	BDNF, HIF1A, IGF1, VEGFA	0.0163
GO.0031102	Neuron projection regeneration	MATN2, OMG, TNC	0.012
GO.0030182	Neuron differentiation	ACTB, ATXN2, HIF1A, HPRT1, KALRN, MATN2, OMG, RHOB, SIAH1, SNAP25, TNC, VEGFA	0.0327
GO.1903376	Regulation of oxidative stress-induced neuron intrinsic apoptotic signaling pathway	FBXW7, HIF1A	0.0159
GO.2000179	Positive regulation of neural precursor cell proliferation	HIF1A, IGF1, VEGFA	0.0368
GO.0001504	Neurotransmitter uptake	SNAP25, SNCA	0.049
GO.0071542	Dopaminergic neuron differentiation	HIF1A, VEGFA	0.049

### Identification of Candidate Novel miRNAs

**FIGURE 8:**
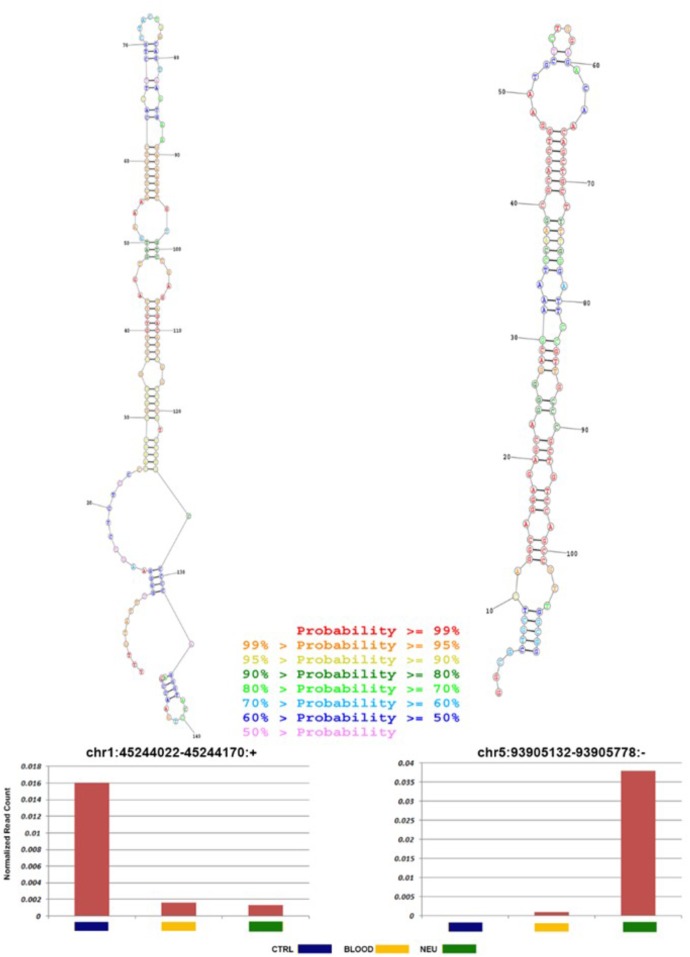
Novel miRNA candidates discovered by next-generation sequencing. Number of reads found for each tissue type and secondary structures of the novel miRNAs. Histograms show the total number of reads sequenced for each sample (normal: blue; ALS blood: yellow; ALS neuromuscular junction: green) and for each portion of the miRNA identified (mature: blue; loop: red; and star: green). All novel miRNAs were computationally predicted to form stem-loop hairpin structures. Putative secondary structures for the two novel miRNAs discovered in this study are shown below the corresponding histograms. Red, yellow, blue, and purple indicate respectively the mature sequence, the loop structure, the predicted star sequence, and the star sequence when identified in our sequencing data.

To foresee novel miRNAs that could have a potential role in ALS pathogenesis and not yet annotated in public databases, we analyzed sequences, which did not map to known mature miRNAs. Preceding the analysis we excluded the reads that aligned against known sncRNAs with the aim to reduce false-positive rate in foreseeing novel miRNAs. miRDeep2 algorithm (Friedländer et al., 2012), which is able to identify novel miRNAs with high precision, was used to analyze the residual reads. Then potential precursor sequences were searched *in silico* and tested for their capacity to form a characteristic hairpin. Using such methods, 49 novel miRNAs, expressed with more than 100 reads in at least three samples, were identified (**Supplementary File S3**). The predicted precursor structure and the sequence of miRNA star corresponding to the novel miRNAs are shown in **Supplementary File S3**. The secondary structure of two novel miRNAs most abundant in ALS blood and neuromuscular junction is shown in **Figure 8**. It is may be observed that the seed regions of the novel miRNAs matched with miRNAs of other species (one mismatch) and are largely conserved among vertebrates, mostly mammals.

## Discussion

It is widely known that involvement of miRNAs is essential to biological processes and their effects on human diseases. The interactions between miRNAs and gene targets are multifaceted, and the expression of miRNAs in different tissues and response to different stimuli is variable depending on genetic background.

The search for novel therapeutic targets has awakened research and placed an increasing importance on miRNAs in their contribution to identifying neurodegenerative diseases.

Amyotrophic lateral sclerosis (Lou Gehrig’s disease) is the most common form of motor disease. Motor neuron diseases (muscle stimulation disorders) are defined by a progressive deterioration of the nerves and other structures involved in the muscular movement. These disorders develop when the motor nerves (which control the muscular movement) do not normally stimulate the muscles. Nerves connect to the muscles at the neuromuscular junction. At this junction, when a nerve excites a muscle, an electrical impulse passes through the muscle triggering contraction.

In motor neuron diseases, motor nerves do not normally stimulate the muscles. As a result, they weaken, they decrease (atrophy) and can paralyze completely. Sporadic ALS (sALS) represents the majority of cases with elusive etiology and only in a small number of cases genetic variations could influence susceptibility to sALS.

In previous researches (De Felice et al., 2012, 2014) we selected, in particular, one miRNA, miR-338-3p. Specific miRNA disease-related upregulation of miR-338-3p, in blood leukocytes as well in serum, spinal cord and cerebrospinal fluid, was detected in sporadic ALS patients.

Since neuromuscular junction is central in this disease, we aimed also to characterize the miRNA expression profile in such tissue using NGS strategy in order to deeply explain sporadic ALS etiopathogenesis and identify novel targets during such disease.

Expression profile of miRNAs was evaluated in neuromuscular junction and blood leukocytes from sALS patients using an NGS platform.

We identified 10 miRNAs highly and differentially expressed in tissues of sALS patients. Of these, we recognized three miRNAs as miR-223-3p, miR-338-3p, miR-342-3p, and miR-326 from neuromuscular junction samples, which could contribute to ALS pathogenesis and were validated by qPCR. In blood leukocytes, we found miR-224-3p and miR 5684 to be overexpressed. In both tissues miR-338-3p was detected highly expressed by qPCR. Previously, we found that such miRNA was highly expressed in several ALS tissue samples as blood, spinal cord, liquor (De Felice et al., 2014).

miR-223-3p is reported as one of the altered miRNA in Huntington’s disease (Díez-Planelles et al., 2016); miR-342-3p has already been found overexpressed in prion induced degeneration (Saba et al., 2008) and miR-326 in multiple sclerosis activity (Zahednasab and Balood, 2014). To evaluate the biological role executed from the aberrantly expressed miRNAs from neuromuscular junction, we performed a functional annotation procedure, obtaining a cohort of 94 gene target associated to ALS. This subset of genes showed a significant enrichment of genes involved in neuronal related pathway (see **Table 1**). Among several pathways, this analysis confirmed the effect of such miRNAs on neurogenesis and neuron death (HIF1A, BDFN1, SNCA, and VEGFA genes), axon guidance and development (BDFN1, RHOB, VEGFA, and KALRN genes), neurotransmitter uptake (SNAP25, SNCA). Several genes as HIF1A and BDFN1, are worthy of further investigation. The brain-derived neurotrophic factor (encoded by BDFN1 gene) as shown in **Figure 5**, is the main node and reported six interactions. VEGFA, ACTB, and KARLN are involved in such pathway. It is well known that kalrin (KARLN gene) indirectly regulates neuronal morphology and growth through the remodeling of the actin cytoskeleton activating several Rho GTPase family members and that its misregulation is associated with neuronal dimorphism, dendritic branching failure and synaptic dysfunction. BDFN1 expression has been found alterated serum levels in subjects with different intensity of cognitive impairment and different neurodegenerative processes (Angelucci et al., 2010; Siuda et al., 2017). Interestingly, in a cohort of serum samples from 45 ALS outpatients (16% bulbar onset), BDNF serum levels were significantly lower in ALS patients expressing lower ALSFRS-R scores (*r* = 0.39, *p* < 0.01), which is a predictor for ALS-progression (Tremolizzo et al., 2016).

Beside, HIF1 pathway has been found significantly perturbed too (FDR = 0.0081). HIF1A, VEGFA, EGLN3, TFRC, and IGF1 essentially contributed to the deregulation of such pathway. HIF-1, hypoxia inducible factor-1, is a transcriptional factor, inducing the expression of genes to enable the survival of cells exposed to hypoxia (Ke and Costa, 2006). Hypoxia is a likely contributor to motor neuron death. Experiments in ALS animal model, SOD1^G93A^ mutant mice, have shown that hypoxia is the main basis of motor neuron death (Tankersley et al., 2007). In cells, hypoxia is counteracted by activating the HIF-1-vascular endothelial growth factor (VEGF) pathway. During hypoxia, VEGF can induce angiogenesis and rises blood quantity to motor neurons (Satriotomo et al., 2016). In ALS patients HIF-1-VEGF pathway is impaired and may contribute to the pathogenesis of the disease, since it has been shown that subjects with compromised response to hypoxia motor neurons are prone to die (Zhang et al., 2011).

Furthermore, we have performed an *in silico* analysis searching for new miRNA candidates. At the moment, high throughput sequencing technologies allows finding novel molecules.

Our analysis used structural alignment against known miRNA families, prediction using iMir and RNAfold, and similarity search against miRNA databases. Of the 49 candidates with any miRNA evidence, two were most abundant in ALS neuromuscular junction and blood respectively. In ALS neuromuscular junction the novel miRNA, chr5:93905132-93905758, was highly expressed compared to control samples with indication of expression of mature sequences. **Figure 7** shows the predicted precursor structure and the sequence of miRNA star corresponding to the novel miRNAs. It may be observed that the seed regions of the novel miRNAs are mostly conserved among mammals and matched with miRNAs of other species (one mismatch). Such data provides a strong probability that these are authentic miRNAs. Additionally, these novel miRNA candidates show a significantly differential expression in all ALS samples (**Figure 7** and **Supplementary File S3**), therefore the role of novel miRNA in ALS needs a deep investigation.

## Conclusion

In conclusion, our deep sequencing analysis has identified novel miRNA signature including different sub-classes of deregulated small RNAs and confirmed miR-338-3p as miRNA expressed in all ALS samples, differentiating normal from neurodegenerative samples. Central evidence on the molecular mechanisms of ALS could be assigned from such data (Bartel, 2004; Filipowicz et al., 2008).

A considerable link between miRNAs deregulation and ALS pathophysiology has been established from impaired miRNAs and target neuronal genes pathways, in particular neuronal intricacy and glutamate uptake capabilities.

This study provides a starting point for future studies to the comprehension of the roles of novel miRNAs in ALS pathological process. Moreover, such small RNAs can be useful biomarkers to support the early and non-invasive diagnosis of ALS.

## Availability of Data and Materials

Data from the LA datasets analyzed during the current study are available from the corresponding author upon reasonable request.

## Ethics Statement

This study was carried out in accordance with the recommendations of Declaration of Helsinki, the institutional review boards of University of Campania “Luigi Vanvitelli”, Italy. The protocol was approved by the institutional review boards of University of Campania “Luigi Vanvitelli”, Italy. All subjects gave written informed consent in accordance with the Declaration of Helsinki.

## Author Contributions

BDF and EB conceived the study. AA and GF collected and analyzed the clinical data and performed the diagnosis. FM and RP performed the experiments. AF analyzed the bioinformatics data. BDF and AF drafted the manuscript. All authors read and approved the final version of the manuscript.

## Conflict of Interest Statement

The authors declare that the research was conducted in the absence of any commercial or financial relationships that could be construed as a potential conflict of interest. The reviewer SP and the handling Editor declared their shared affiliation.

## Footnotes

1 www.ebi.ac.uk/arrayexpress

## Supplementary Material

TABLE S1 General characteristics of the ALSs patients.

FILE S1 Length distribution of clean reads. Length distribution of reads aligned to miRNAs: the aligned read length ranges from 17 to 27 nucleotides with the maximum distribution of reads captured between 18 and 25 nucleotides (reflecting miRNA read length).

FILE S2 Fold change and posterior probability of miRNAs in ALS blood and neuromuscular junction samples.

FILE S3 List of novel miRNAs in ALS samples reporting the coordinate of pre-miRNA, the pre-miRNA sequence and mature sequence.

## Abbreviations

ALS: amyotrophic lateral sclerosis
miRNA: microRNA, small non-coding RNA molecule
NGS: next-generation sequencing
ORF: open reading frame
qRT-PCR: quantitative reverse transcription polymerase chain reaction
sALS: sporadic amyotrophic lateral sclerosis
UTR: untranslated region
