# Supplementary material for: Wide-Ranging Analysis of MicroRNA Profiles in Sporadic Amyotrophic Lateral Sclerosis Using Next-Generation Sequencing
Source: Front Genet. 2018 Aug 14;9:310. doi: 10.3389/fgene.2018.00310 (PMC6102490; doi:10.3389/fgene.2018.00310)
Supplement: TABLE S1 — General characteristics of the ALSs patients. [file Table_1.DOCX]

**Table1. General characteristics of the ALSs patients**

| **Parameter** | **ALS patients** |
| --- | --- |
| Female, n (%) | 25 (45) |
| Median onset of ALS (years)^1^ | 65.0 (40–70) |
| Bulbar/Spinal | 19/45 |
| Median duration of ALS at examination, range (months) | 18.5 (12–25) |
| Median ALS-FRS-R Score, range | 26.0 (6–46) |
