# Supplementary material for: Wide-Ranging Analysis of MicroRNA Profiles in Sporadic Amyotrophic Lateral Sclerosis Using Next-Generation Sequencing
Source: Front Genet. 2018 Aug 14;9:310. doi: 10.3389/fgene.2018.00310 (PMC6102490; doi:10.3389/fgene.2018.00310)
Supplement: FILE S3 — List of novel miRNAs in ALS samples reporting the coordinate of pre-miRNA, the pre-miRNA sequence and mature sequence. [file Presentation_1.PDF]

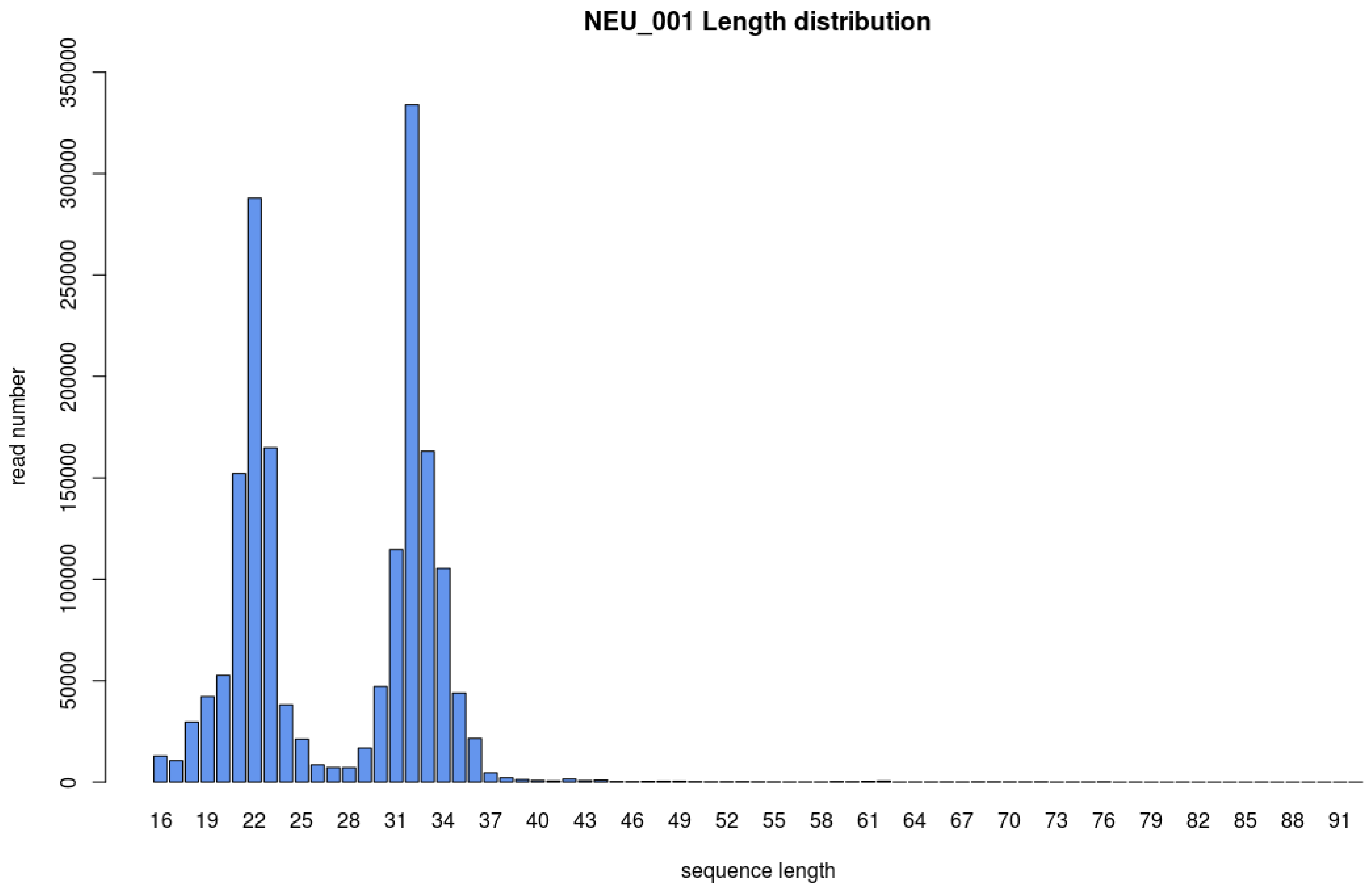
NEU_001 Length distribution
read number
0
50000
100000
150000
200000
250000
300000
350000
16 19 22 25 28 31 34 37 40 43 46 49 52 55 58 61 64 67 70 73 76 79 82 85 88 91
sequence length

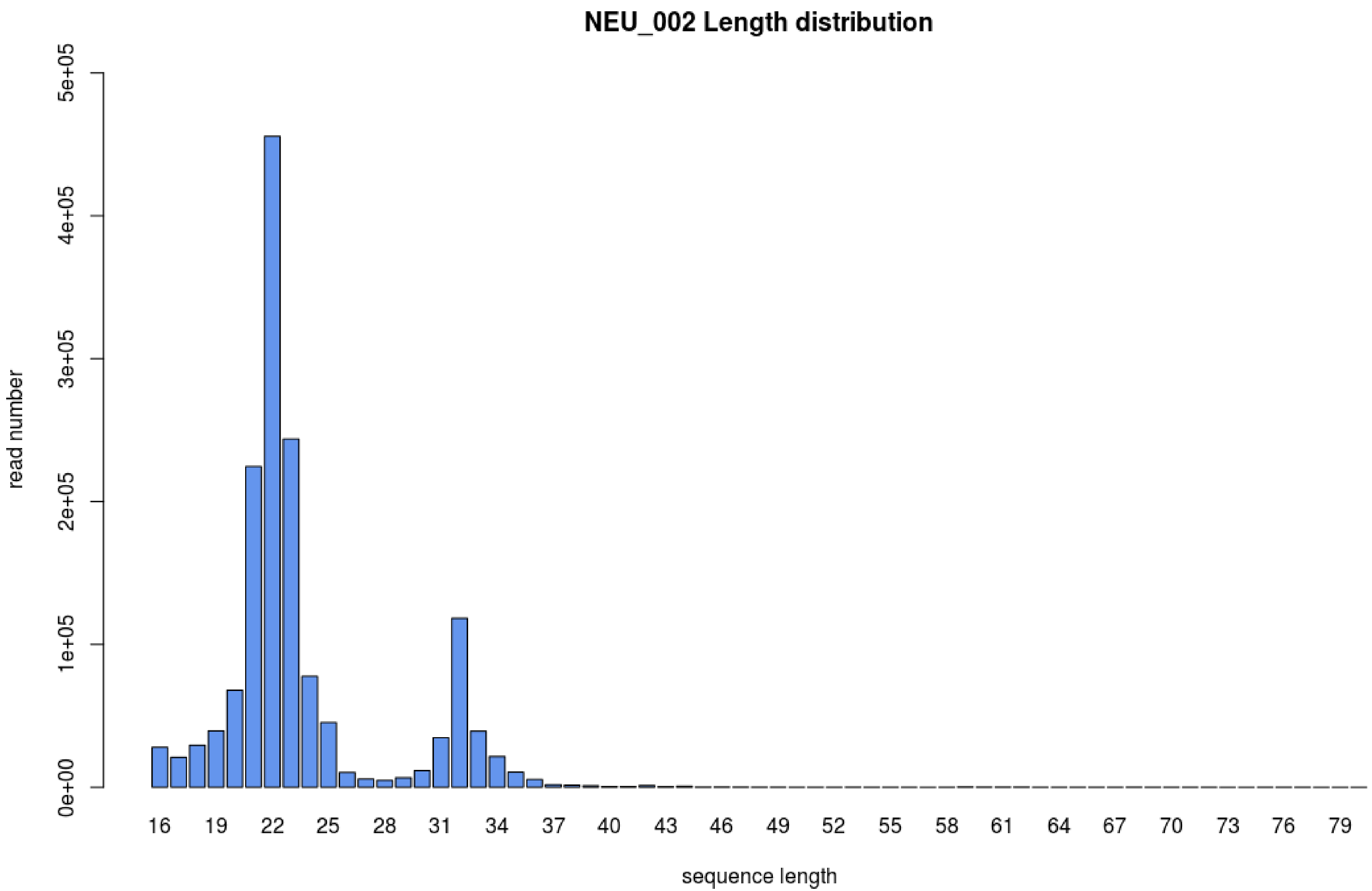

NEU_002 Length distribution
read number
0e+00
1e+05
2e+05
3e+05
4e+05
5e+05
16
19
22
25
28
31
34
37
40
43
46
49
52
55
58
61
64
67
70
73
76
79
sequence length

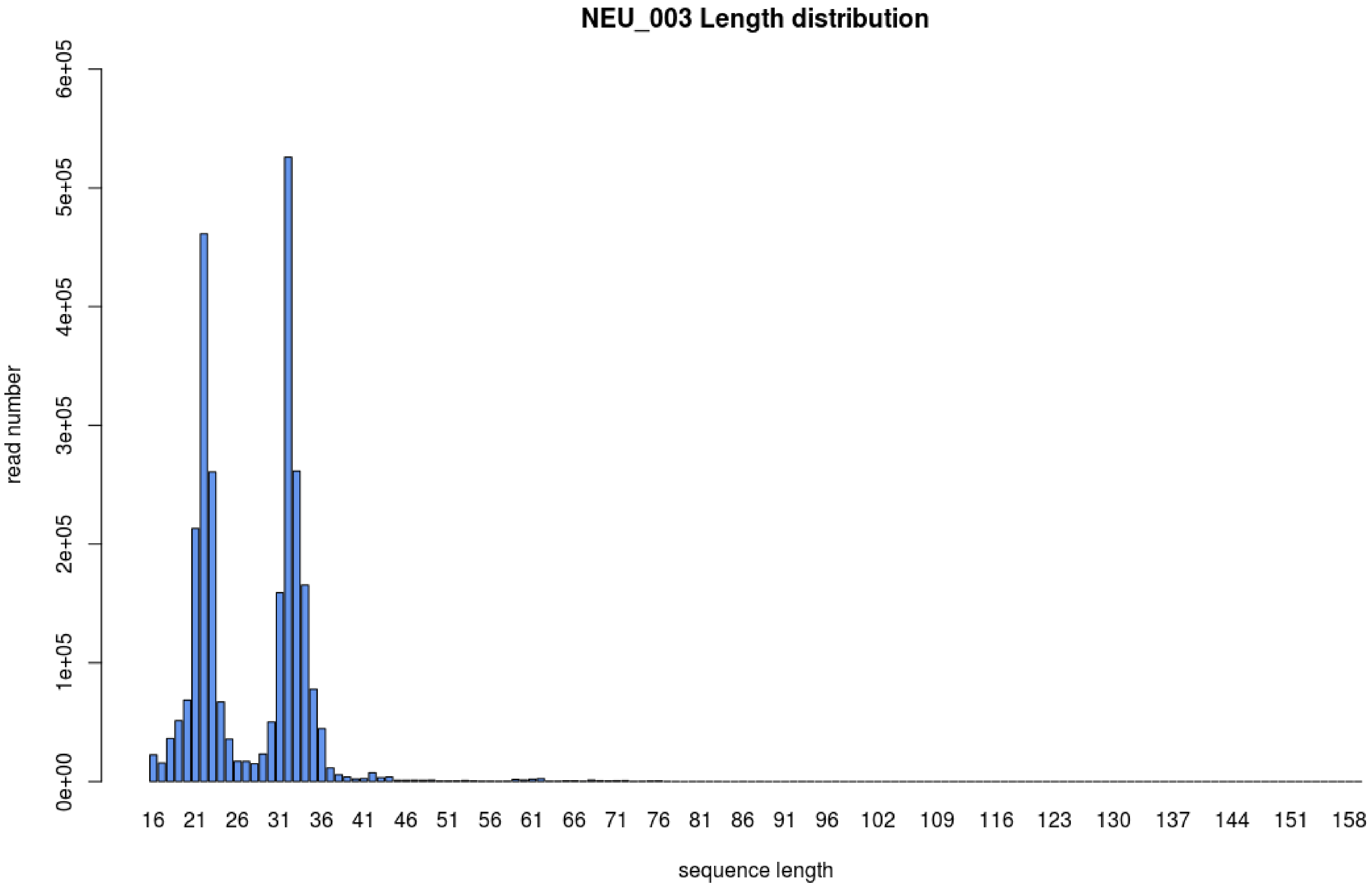
NEU_003 Length distribution
read number
0e+00
1e+05
2e+05
3e+05
4e+05
5e+05
6e+05
16
21
26
31
36
41
46
51
56
61
66
71
76
81
86
91
96
102
109
116
123
130
137
144
151
158
sequence length

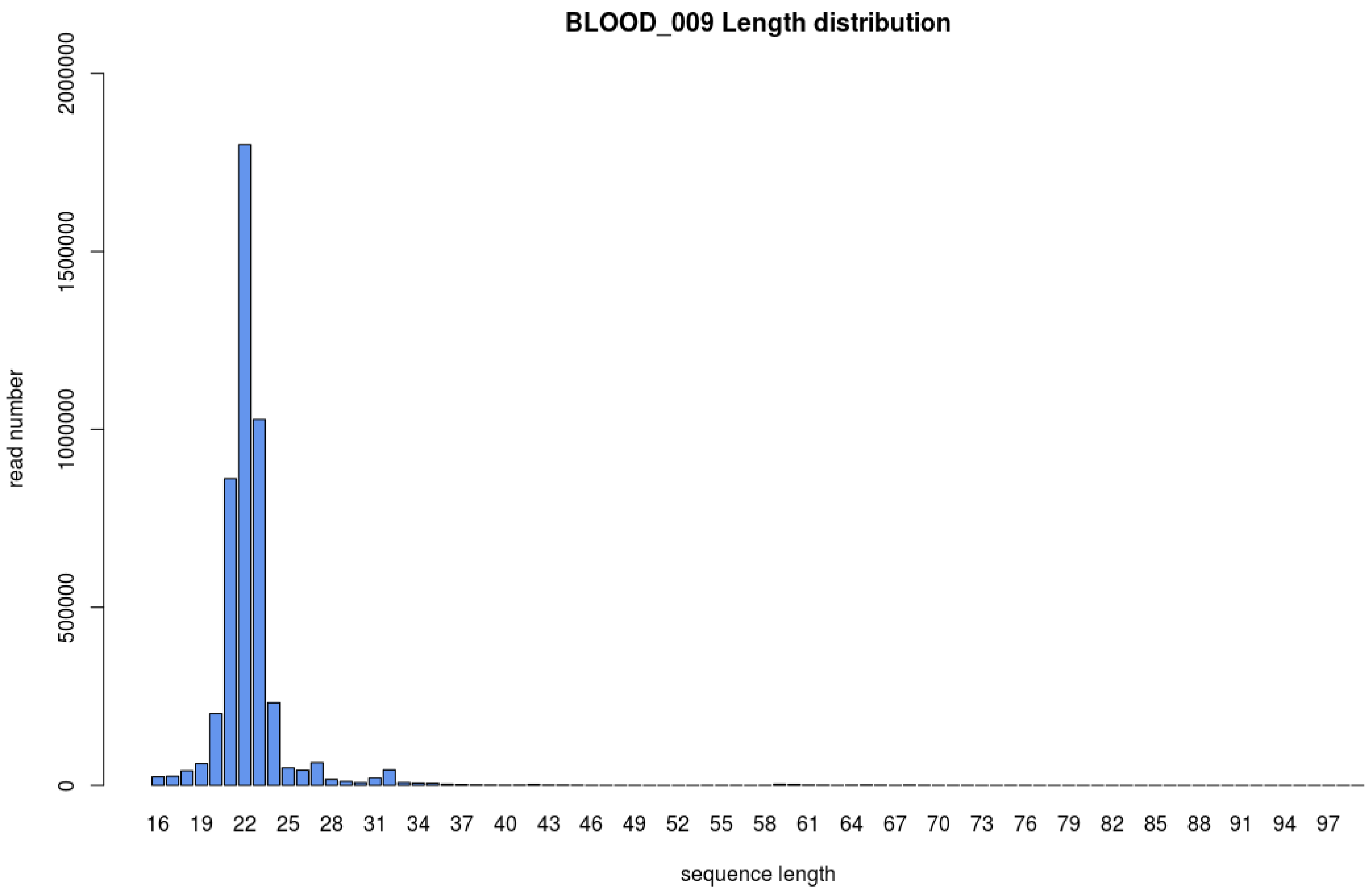

BLOOD_009 Length distribution
read number
0
500000
1000000
1500000
2000000
16
19
22
25
28
31
34
37
40
43
46
49
52
55
58
61
64
67
70
73
76
79
82
85
88
91
94
97
sequence length

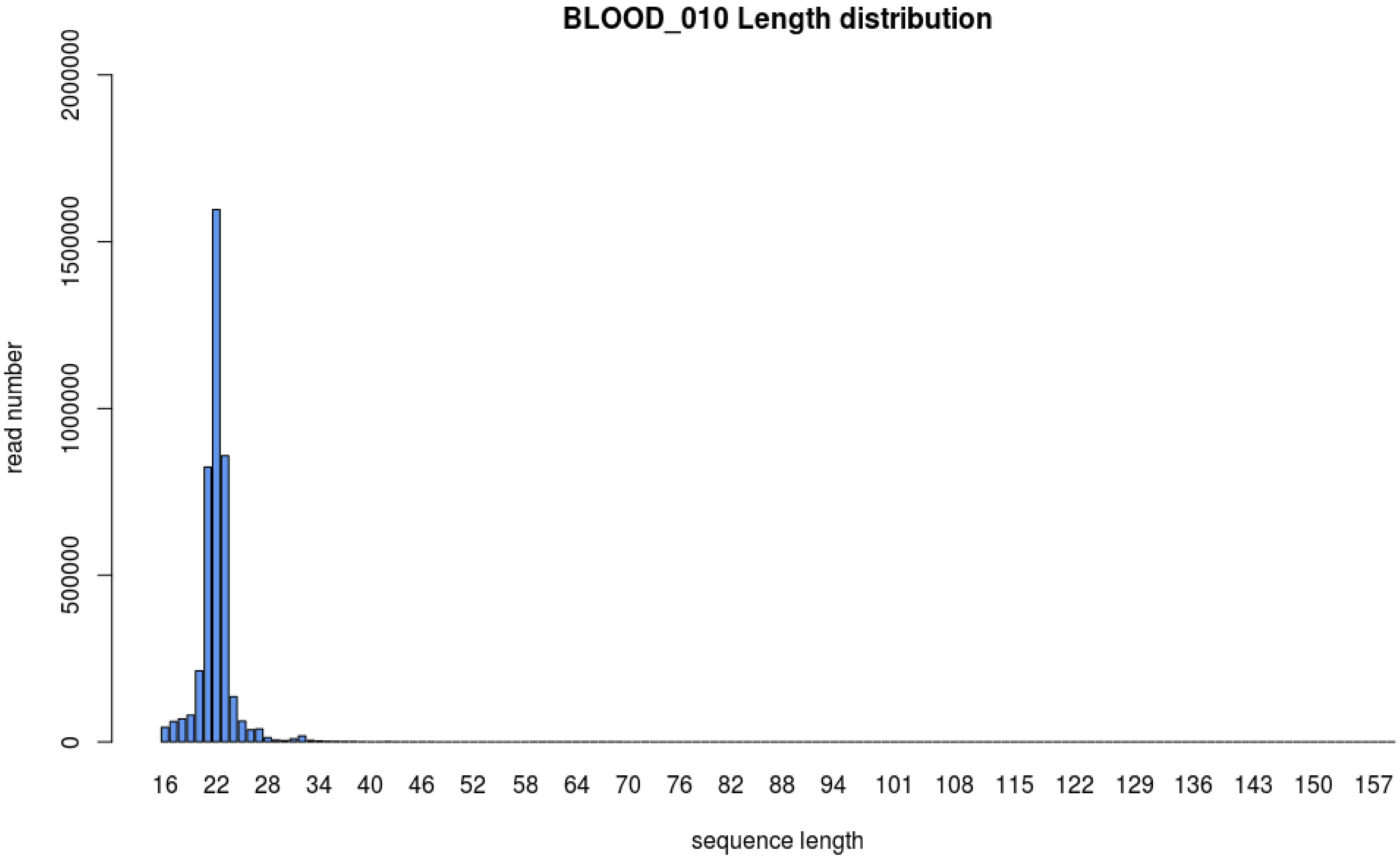
BLOOD_010 Length distribution
read number
0
500000
1000000
1500000
2000000
16 22 28 34 40 46 52 58 64 70 76 82 88 94 101 108 115 122 129 136 143 150 157
sequence length

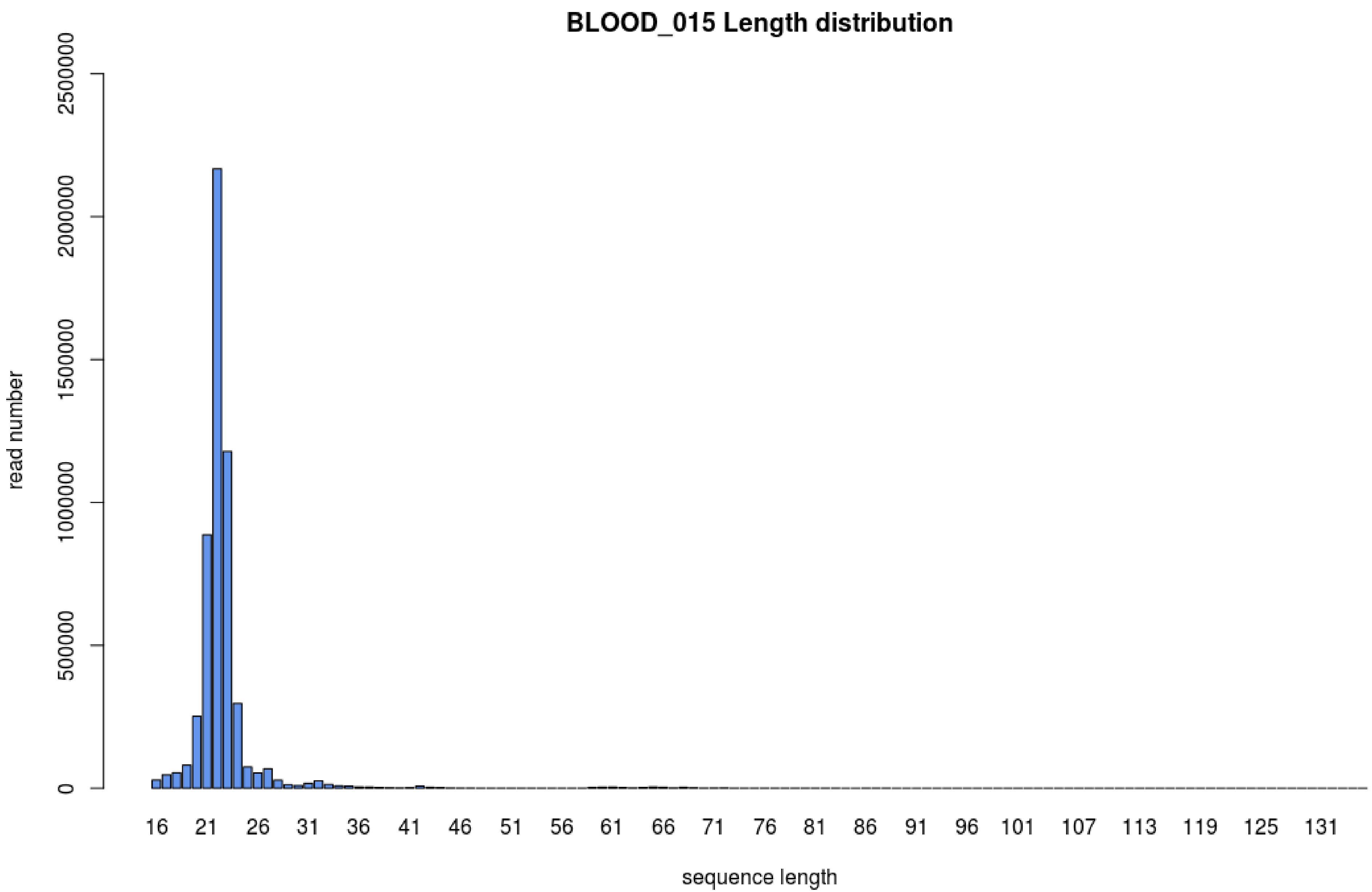

BLOOD_015 Length distribution
read number
2500000
2000000
1500000
1000000
500000
0
16 21 26 31 36 41 46 51 56 61 66 71 76 81 86 91 96 101 107 113 119 125 131
sequence length

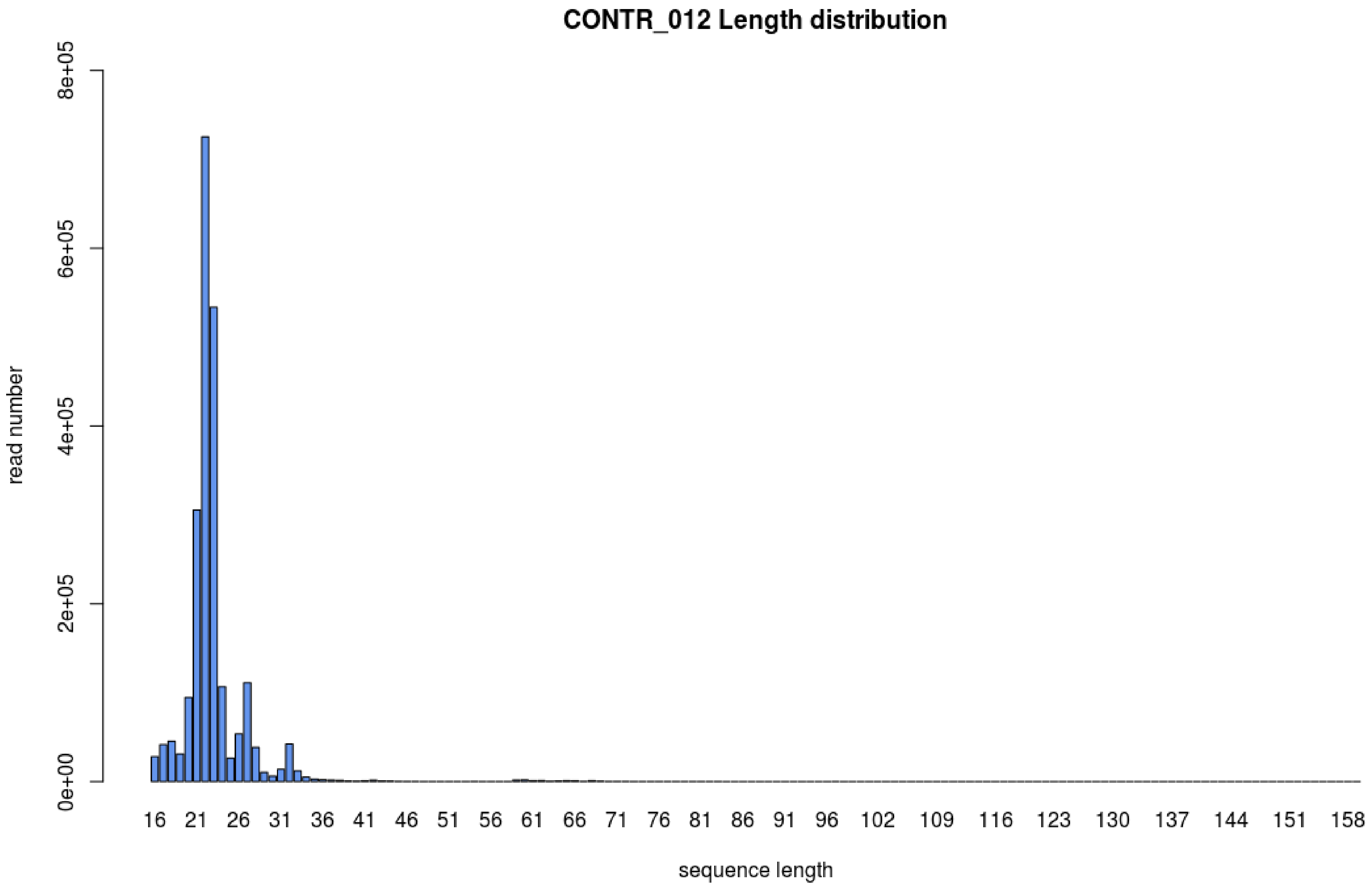
CONTR_012 Length distribution
read number
0e+00
2e+05
4e+05
6e+05
8e+05
16
21
26
31
36
41
46
51
56
61
66
71
76
81
86
91
96
102
109
116
123
130
137
144
151
158
sequence length

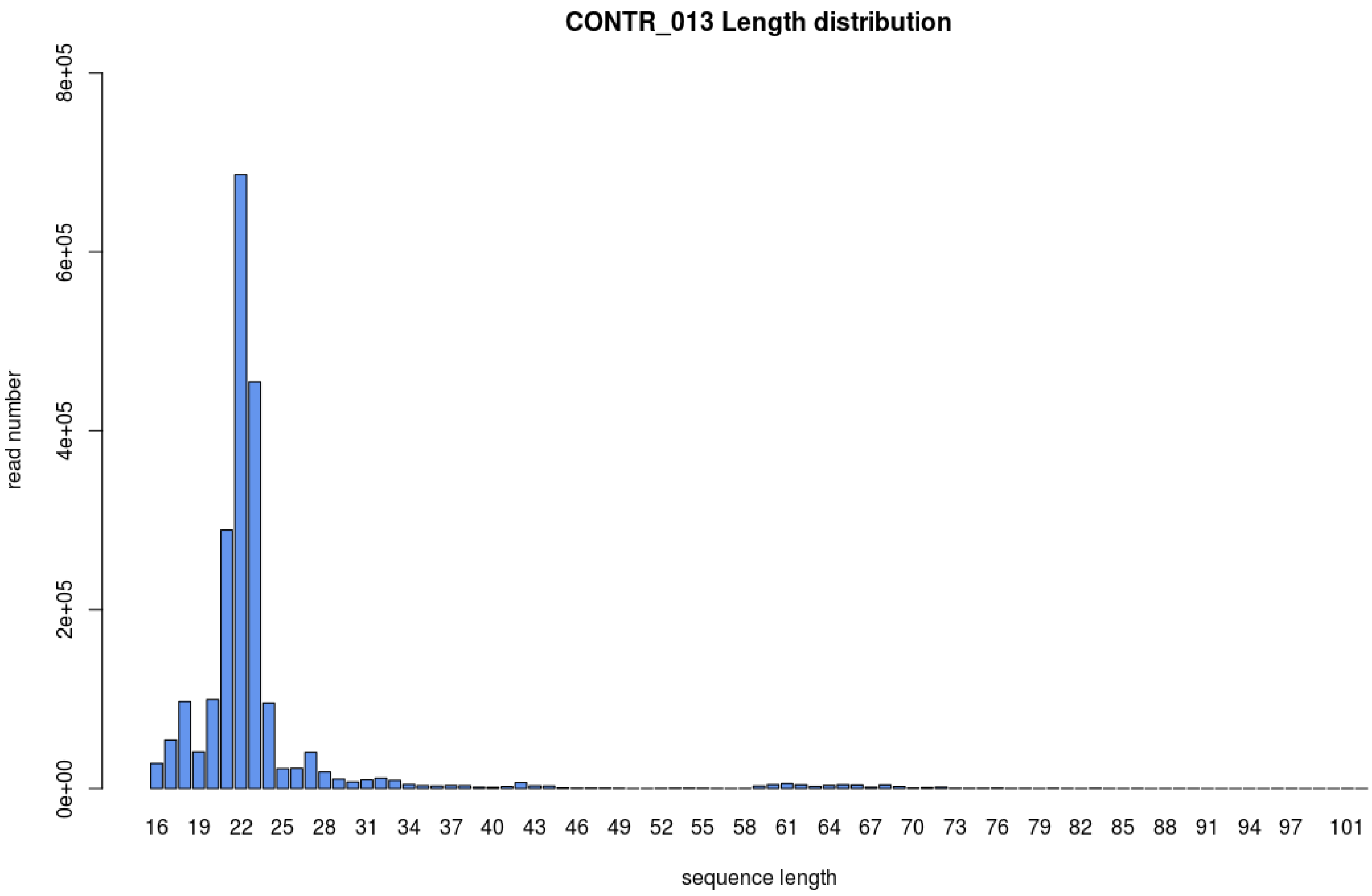
CONTR_013 Length distribution
read number
0e+00
2e+05
4e+05
6e+05
8e+05
16 19 22 25 28 31 34 37 40 43 46 49 52 55 58 61 64 67 70 73 76 79 82 85 88 91 94 97 101
sequence length
